# Influence of Seed-Covering Layers on Caper Seed Germination

**DOI:** 10.3390/plants12030439

**Published:** 2023-01-18

**Authors:** María Laura Foschi, Mariano Juan, Bernardo Pascual, Nuria Pascual-Seva

**Affiliations:** 1 Departamento de Producción Vegetal, Universitat Politècnica de València, 46022 Valencia, Spain; 2 Horticulture and Floriculture, Agriculture Faculty, National University of Cuyo, Mendoza M5528AHB, Argentina; 3 Centro Valenciano de Estudios sobre el Riego, Universitat Politècnica de València, 46022 Valencia, Spain

**Keywords:** abscisic acid, gibberellins, micropylar endosperm, puncture force, seed dormancy

## Abstract

Caper is a perennial shrub that is widespread in the Mediterranean Basin. Although the fruits contain many seeds, they germinate slowly and with very low percentages, due to their nondeep physiological dormancy. The influence of the testa and endosperm, as well as the effect of applying gibberellic acid (GA_3_) solutions on seed germination to release its dormancy, are reported in this study. The mechanical resistance exerted by the testa and endosperm against radicle protrusion in mature caper seeds was measured. The best germination results were obtained with seeds devoid of testa wetted with water and with intact seeds wetted with a 500 mg L^−1^ GA_3_ solution, without statistical differences between them. The GA_3_ addition triggers an increase in both the content of endogenous gibberellins (GA) and the GA/abscisic acid ratio, increasing germination. Its germination consists of two temporally separated events: testa cracking and endosperm piercing. Testa cracking begins in the hilum-micropillar area; it involves a signal from the embryo, which GA can replace, possibly by increasing the growth potential of the embryo. After testa cracking, the radicle emerges through a hole in the micropylar endosperm. The puncture force necessary to pierce the micropylar endosperm decreased drastically during the first day of imbibition, remaining practically constant until testa cracking, decreasing afterwards, regardless of the addition or not of gibberellins.

## 1. Introduction

The caper (*Capparis spinosa* L.) is a perennial shrub that is naturally widespread throughout the Mediterranean Basin in traditional and specialized systems for commercial production [1]. It is mainly cultivated in arid environments in Morocco, Turkey, the Pantelleria and Salina Islands in Italy and the Balearic Islands in Spain, as well as on the southeastern Iberian Peninsula [2]. It has a great variety of uses, having a high agricultural potential, particularly in food (principally for its flower buds and semimature fruits, pickled in brine) and in the pharmaceutical (to prevent cardiovascular and gastrointestinal diseases) and cosmetic industries [3]. Some researchers have proposed the use of nonedible caper seed oils to obtain biodiesel as an alternative and renewable energy source, which could contribute to lowering the impact on the environment, thus reducing the effects of climate change by reducing the use of fossil fuels [4,5]. Given the resistance of caper to drought and its ability to reduce soil erosion, in the Mediterranean Basin, it is cultivated in intercrops with cereals, vines and both olive and almond trees [6] and is of particular interest on marginal arid lands. Furthermore, drought resistance and its ornamental value make caper an interesting plant to be used in xerogardening and landscaping [3].

**Figure 1:**
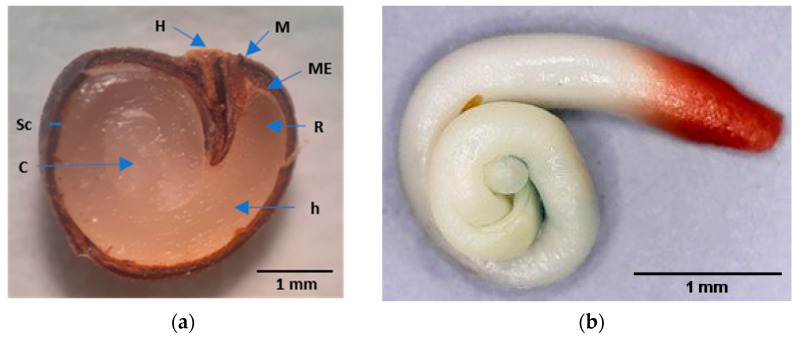
Caper seed. (**a**) Longitudinal section of a mature caper seed after soaking in water for four days. H, hilum; M, micropyle; ME, micropylar endosperm; R, radicle; h, hypocotyl; Sc, seed coat; and C, cotyledons; (**b**) Embryo with a stained radicle with tetrazolium.

This plant has developed mechanisms to survive in Mediterranean conditions [2], including the already mentioned tolerance to drought and seed dormancy. Although the fruits contain many (more than 150 seeds fruit^−1^) small (with an average maximum Feret diameter of 3.3 mm) reniform and dark-brown seeds (Figure 1), they have a very low germination percentage.

The caper seed coat is bitegmic, formed by testa and tegmen, being impermeable and hard to the touch. Its thickness is approximately 0.2 mm, and it has two anatomical structures, the hilum and micropyle (Figure 1). The testa is formed by a layer of one to two thick cells with very lignified and thickened walls; the external epidermis has stomata [7]. The teg-men consists of a lignified exotegmen composed of a layer of several brachysclereid cells and a lignified, fibrous endotegmen composed of a few layers of cells. The set of testa and tegmen are referred to simply as testa in this paper. The endosperm comprises several cell layers surrounded by a tightly embedded cuticle. The embryo has a long, robust, coiled hypocotyl, a short radicle and coiled cotyledons (Figure 1).

The germination process starts with water uptake by the dry seed, which is followed by embryo expansion growth and concludes with the rupture of the covering layers and emergence of the radicle, which is considered the completion of germination [8]. Under optimal supply conditions, the typical uptake of water by seeds is triphasic [9]. There is initially a phase of rapid water absorption (Phase I), followed by a second phase (Phase II or lag phase) in which the plant stabilizes and begins the activation of metabolism and the mobilization of nutrients. Seeds that complete germination enter the third phase (Phase III), which begins with radicle protrusion.

Dormancy is the temporary failure of seeds to germinate under favorable conditions. There are different types of dormancy: (i) physical, mechanical, or chemical inhibition caused by the covering layers of the embryo; (ii) the inability to germinate because of an undifferentiated or immature embryo; and (iii) germination repression originating from metabolic restrictions [9]. Based on the Nikolaeva system to classify dormancy types, Baskin and Baskin [10] proposed a hierarchical classification system that includes five classes of seed dormancy: physiological (PD), morphological (MD), morphophysiological (MPD), physical (PY) and combinational (PY + PD). According to these authors, water-impermeable layers of palisade cells in the seed that control water movement cause PY. This could be the case for caper seeds, given that their testa structure could affect their dormancy persistence [11,12], which the authors of [2] also hypothesized, pointing to the caper seed coat as one of the causes of the low germination rate. However, our research team [3,12] stated that the lack of imbibition is not a determining factor for the germination of caper seeds, since seed hydration begins through the hilum-micropylar region until reaching the endosperm and the embryo; therefore, these seeds do not present PY due to the impermeability of the seed coat.

To obtain high germination percentages, the use of gibberellins (GA) is required [13,14], which suggests the presence of PD in the embryo by requiring a softening of the seed-covering layers (the testa and endosperm) and/or an increase in force or potential of embryo growth to achieve seed germination. PD is the most abundant form of dormancy found in seeds of the major angiosperm clades [15]. PD has been divided into three levels: deep, intermediate and nondeep [10].

According to the internal morphology of the embryo and endosperm in mature angi-osperm seeds, caper seeds belong to the FA-2 seed type [16]. Some species within this type, including *Arabidopsis thaliana*, have nondeep PD [15]. Seeds that have nondeep PD may produce normal seedlings from embryos excised from seeds; depending on the species, GA can release this dormancy, which can also be released by scarification, after-ripening in dry storage and cold or warm stratification. Hormones play an essential role in dormancy status, maintaining or releasing it. Although other dormancy-releasing chemicals (ethylene, cytokinin, auxin, brassinosteroids, nitrogenous compounds and butenolides) exist, GA and abscisic acid (ABA) profoundly influence seed dormancy and germination. This influence is affected during the lag phase of germination by hormone biosynthetic and catabolic enzymes, whose abundance is controlled primarily at the transcriptional level [9].

The embryo-covering layers in seeds that have PD can confer a mechanical restriction (coat dormancy) that the embryo growth potential must overcome [9]. The success of germination in seeds with PD-type coat dormancy demands that the embryo growth potential increases to overcome the mechanical restriction and/or that the mechanical re-striction associated with the seed-covering layer(s) is reduced. These two mechanisms are known as ‘embryo dormancy’ and ‘coat dormancy’, respectively [15], being both components of PD and determined by their sum and interaction, the degree of the ‘whole-seed’ PD. Embryo dormancy inhibits extension growth; thus, excised embryos do not grow. Non-dormant embryos excised from coat-dormant seeds can therefore extend and grow. ‘Coat’ is used loosely, referring to any embryo-covering structure, such as the testa and/or endosperm. The testa consists mainly of dead tissues, and it is the seed’s interface with the external environment, protecting the embryo against adverse environmental conditions; it imposes a mechanical restriction in coat-imposed seed dormancy that controls germination timing [17]. In many species, such as caper, a living layer of endosperm is interposed between the testa and the embryo; endosperm weakening occurs before germination, and the tissue can produce enzymes for initiating this process [15].

The main aim of the present study was to determine the influence that the testa and the endosperm exert against radicle protrusion, the influence of these covering layers and the influence of an application of a gibberellic acid (GA_3_) solution on seed germination. Therefore, two germination tests were carried out using seeds with and without testa (consisting of endosperm and embryo), wetting the substrate with water and a GA_3_ solution. The hormone (GA and ABA) content of caper seeds was determined in different phases of the germination process. The mechanical resistance that the testa and endosperm exert against radicle protrusion in dry, mature caper seeds was also determined by measuring the puncture force necessary to perforate the testa and endosperm, which was determined from the beginning of imbibition until radicle protrusion.

## 2. Results and Discussion

### 2.1. Viability and Germination Tests

The viability of the seeds was 87.5% ± 4.8%, similar to that obtained in other lots of caper seeds by the research team [3,18], who related the high viability to the fact that the fruits were collected at their physiological maturity and to the careful extraction, cleaning, handling and storage of the seeds.

**Figure 2:**
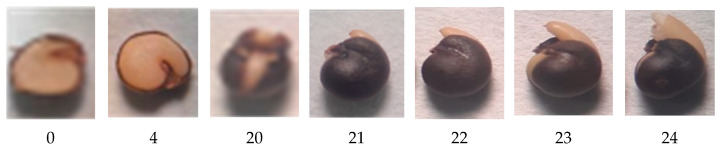
From left to right, photographs correspond to the number of days elapsed since the start of the germination test (Day 0; dry mature seed) through to the opening of the testa (Day 20) and elongation of the hypocotyl and radicle inside the endosperm (from Day 20 to 23), until the rupture of the micropylar endosperm (Day 24). On Days 0 and 4, half of the testa was removed to observe the state of the endosperm.

Caper seed germination consists of two temporally separated events: first, testa cracking occurs, starting at the hilum-micropylar area and requiring water absorption and swelling of the embryo and endosperm, and then rupture of the endosperm occurs (Figure 2), as stated by Moghaddasi Mohammad et al. [19]. These two events also occur in many seeds of the Solanaceae (as *Nicotiana tabacum* [15]) or Brassicaceae (as *Lepidium sativum* and *Arabidopsis thaliana* [20]) families. These two events are mechanically distinct processes [15], because, as previously indicated, the testa is dead in these species, and the endosperm is living tissue. 

**Figure 3:**
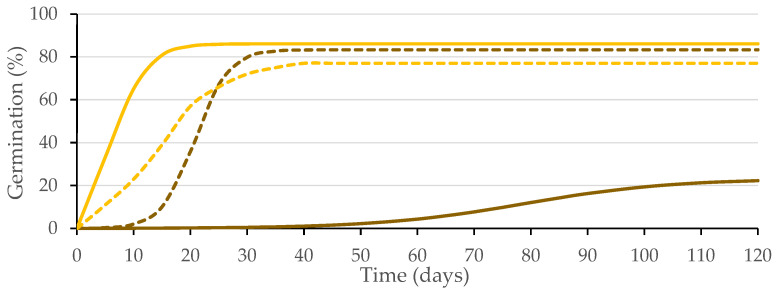
Logistic model adjusted to the germination curves made for seeds with intact testa (brown) and without testa (yellow) with substrate wetted with water (solid line) or GA_3_ solution (500 mg L^−1^; dashed line). Average values of four replicates.

**Figure 4:**
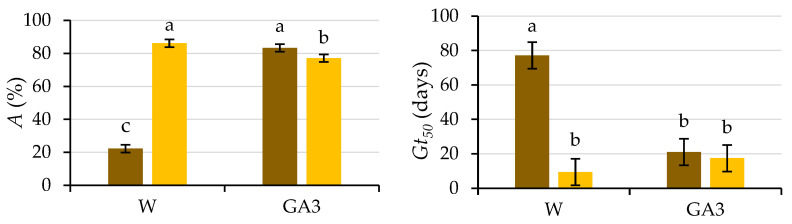
Analysis of the significant interactions of the analysis of variance in Table 1 between the presence of the testa (with testa (brown) and without testa (yellow)) and the solution used to wet the substrate (water (W) or GA3) on the final germination (*A*) and the number of days needed to reach 50% of the final germination (*Gt*_50_). Average values of four replicates. Different letters indicate significant differences according to the LSD test. Error bars represent the LSD (*p* ≤ 0.05).

The coefficients of determination (R^2^) obtained for the germination data fitted to the logistic function (*p* ≤ 0.01) for each replicate from the four combinations of variation sources were greater than 0.94 (Figure 3), allowing the use of the variable *A* (instead of *G*), as well as other variables, such as *Gt*_50_ and *k*/2, as performed in previous studies of caper seed germination [14,21]. Significant differences were obtained (*p* ≤ 0.01; Table 1) for the presence of testa and the solution used, as well as for their interaction (*p* ≤ 0.01; Table 1). Given that this interaction explains 44% of the variability of the test, it is presented in Figure 4.

The difference obtained between the germination of seeds with (22%) and without testa (86%, i.e., all viable seeds germinated) when water was used to wet the germination substrate suggests coat-imposed dormancy due to mechanical characteristics, since previous studies [3] have ruled out insufficient seed coat permeability. In the case of coat-imposed dormancy, removal of the tissues surrounding the embryo (e.g., testa and/or endosperm) in some species (in this experiment, the testa) is enough for successful completion of germination [15,22]. However, the testa-removal procedure is very laborious, and it may damage the embryos [23]; therefore, in some studies, such as those carried out in caper seeds [11,24,25], only an incision in the seed coat was made with a scalpel. Our results are in line with those obtained by Sozzi and Chiesa [11], who performed surgical treatment on caper seeds that had failed to respond to dormancy-breaking pretreatments to improve the germination of caper seeds, performing an incision with a scalpel close to the radicle. With this piercing, 100% of viable embryos germinated within 3–4 days, being released from seed dormancy. The results presented herein also agree with the high germination percentages obtained by Chalak et al. [24] for dormancy-breaking treatment for in vitro propagation of caper seeds whose coat was scarified with a scalpel (up to 71%). Elazazi [25] achieved the highest germination percentage (98%) and the fastest germination using mechanical scarification in freshly collected caper seeds. When the seed coat is damaged (cracked or removed), the force it imposes is sufficiently reduced to no longer be a constraint on the low growth potential of the embryo [26].

The seeds devoid of the testa germinated earlier (*p* ≤ 0.05), both in water (*Gt*_50_ = 9.4 d) and in the GA_3_ solution (*Gt*_50_ = 17.5 d), than those intact seeds germinated in water (*Gt*_50_ = 77.1 d). The *Gt*_50_ for seeds with testa wetted with GA_3_ (*Gt*_50_ = 21 d) was lower (*p* ≤ 0.05) than those wetted with water, not differing (*p* ≤ 0.05) from the time required by the seeds without the testa (Figure 4).

**Figure 5:**
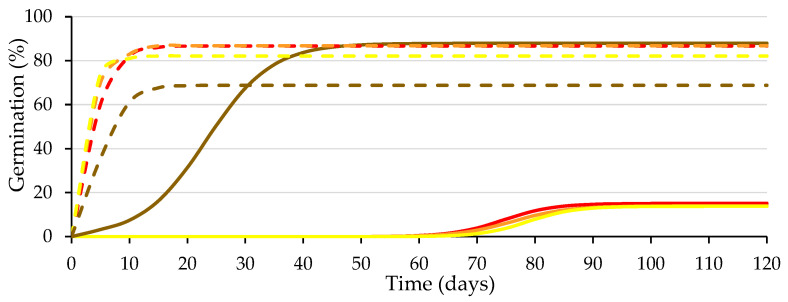
Logistic model adjusted to the germination curves of the seeds with (solid lines) and without testa (dashed lines) with substrate wetted with different GA_3_ concentration solutions (0, 5, 50 and 500 mg L^−1^ in red, orange, yellow and brown, respectively). Average values of four replicates.

Considering the lower germination achieved in seeds devoid of the testa wetted with the GA_3_ solution (77%) than that achieved with water (86%), on 1 July 2021, a new germination test was started with seeds belonging to the same lot to analyze the response of the seeds to different GA_3_ concentrations (0; 5; 50 and 500 mg L^−1^) used for wetting the substrate. Figure 5 presents the logistic model adjusted to the different treatment combinations, and Table 2 presents the statistical analysis of the germination parameters. The difference between the germination values obtained in the two tests does not exceed the tolerance established by International Rules for Seed Testing (ISTA) [27].

**Figure 6:**
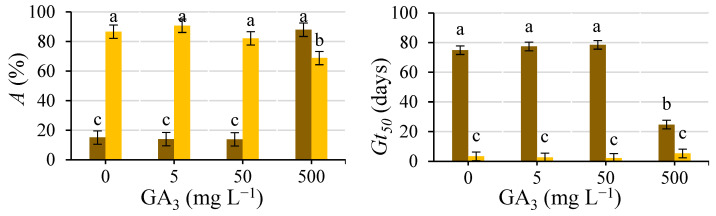
Analysis of the significant interactions of the analysis of variance in Table 2 between the presence of the testa (with testa (brown) and without testa (yellow)) and the concentration of the GA_3_ solution used to wet the substrate (0, 5, 50 and 500 mg L^−1^) on *A* and *Gt*_50_. Average values of four replicates. Different letters indicate significant differences according to the LSD test. Error bars represent the LSD (*p* ≤ 0.05).

The presence–absence of testa explains 52% (*p* ≤ 0.01) of the total variability for *A*. Its interaction with the GA_3_ concentration of the wetting solution explains 33.6% (*p* ≤ 0.01), and it is presented in Figure 6.

Regarding intact seeds, the only GA_3_ concentration that improved (*p* ≤ 0.05) germination compared to the control was the 500 mg L^−1^ solution, increasing (*p* ≤ 0.05) *A* and decreasing (*p* ≤ 0.05) *Gt*_50_, with the lower concentrations being inefficient, which agrees with previous studies carried out by our research team [28]. For the seeds devoid of the testa, the GA_3_ addition did not improve (*p* ≤ 0.05) germination compared to water, even decreasing *A* (*p* ≤ 0.05) by 500 mg L^−1^.

Müller et al. [20] extracted the embryos from the endosperms of the seeds of *Lepidium sativum* and obtained an interaction between the embryo and endosperm during a time frame between 2 and 5 h that could induce endosperm weakening by a signal exercised by the embryo; after this period, this weakening did not require the presence of the embryo. They stated that the embryo signal could be replaced by GA, causing the complete weakening of micropylar endosperm (ME) isolated at 2 h. Similarly, caper seeds may not need the imbibition of the GA_3_ solution throughout the germination test, as demonstrated in several studies in which statistically greater germination percentages were obtained with caper seeds soaked for 12–24 h in GA_3_ solutions than in control seeds and similar to those obtained by wetting the germination substrate throughout the germination test [28]. The seeds devoid of testa did not need the imbibition of GA_3_; in addition, keeping these seeds devoid of testa wet throughout the test using a solution of such a high concentration of GA_3_ could cause a decrease in the germination rate (Figure 6), which could be considered as an onset of AG_3_ toxicity [29,30,31].

### 2.2. Hormone Content

**Figure 7:**
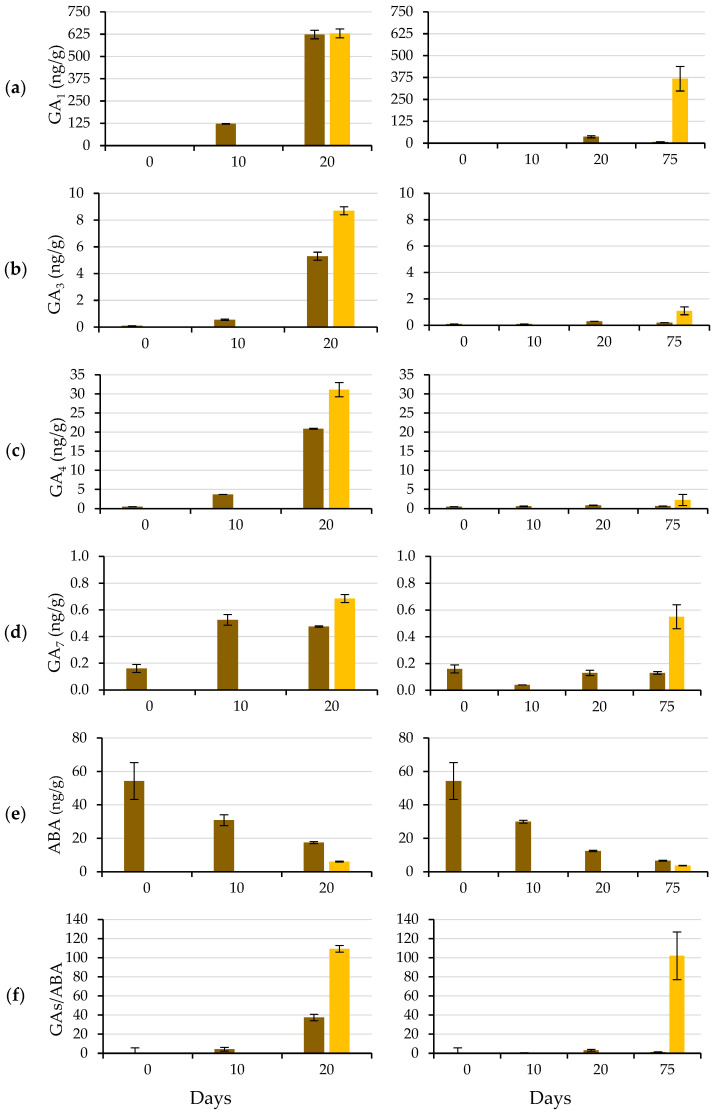
GA_1_ (**a**), GA_3_ (**b**), GA_4_ (**c**), GA_7_ (**d**) and ABA (**e**) contents and the GAs/ABA ratio (**f**) at 0, 10, 20 and 75 (if applicable) days after of the start of the germination test, using a GA_3_ (500 mg L^−1^) solution (left) or water (right) to wet the substrate. Day 20 (75) corresponds to the protrusion of the radicle of the seeds wetted with GA_3_ (water). Non-germinated seeds in brown, and germinated seeds in yellow. Mean values ± SE of 40 seeds (two replicates of 20 seeds).

Figure 7 shows the time course of GA_1_ (a), GA_3_ (b), GA_4_ (c), GA_7_ (d) and ABA (e) contents and the GA/ABA ratio (f). The content of GA, especially that of GA_1_, increased with the germination process, whereas the concentration of ABA decreased. This increase in GA content was faster when the GA_3_ solution was used than when water was used to wet the substrate, whereas the decrease in ABA content was similar in both cases.

Exogenous GA_3_ applications increased the contents of endogenous bioactive GA, particularly GA_1_, agreeing with Yuxi et al. [32], who reported that GA_1_ was the most abundant GA after applying GA_3_ to peony plants. The increasing content of endogenous GA was probably due to de novo biosynthesis rather than a substantial conversion of GA_3_ to GA_1_ and GA_4_ (Servicio de Cuantificación de Hormonas Vegetales, personal communication). These increasing contents of endogenous GA probably regulate GA biosynthesis and signal transduction through feedback [32].

Bioactive GA (such as GA_1_ and GA_4_) are biosynthesized from geranylgeranyl diphosphate (GGDP) via a three-step process: (i) synthesis of ent-kaurene from GGDP; (ii) conversion of ent-kaurene to GA_12_; and (iii) synthesis of 19- and 20-carbon GA from GA_12_. In the third step, the process can mainly follow two pathways, which vary according to the species [33,34]: GA_4_ is synthesized through the non-13-hydroxylation pathway, and GA_1_ is synthesized through the early 13-hydroxylation pathway. Although GA_4_ exists in most species and is thought to be the main bioactive GA in *Arabidopsis thaliana* and some Cucurbitaceae members [33], GA_1_ is the major bioactive form in other species, as occurs in caper. Most likely, the early 13-hydroxylation pathway is the predominant synthesis pathway in caper seed germination.

As Bewley et al. [9], stated, there is coregulation or ‘cross-talk’ between GA and ABA, i.e., ABA regulates GA metabolism and signal transduction, and GA affect ABA metabolism and signal transduction reciprocally. This coregulation implies that each signal is amplified rapidly (in this case, GA_3_ triggers an increase in GA content and response by eliminating ABA production and signal transduction). ABA determines seed dormancy and inhibits seed germination, and GA can release nondeep PD. The GA/ABA balance is as important as the hormone levels for dormancy release [35,36,37]. Indeed, during the germination process, the GA/ABA balance increased, reaching values greater than 100 in the newly germinated seeds (start of Phase III), both when the GA_3_ solution was used (at Day 20) and when water was used (at Day 75). In addition to the hormone content, the transition from the nondeep PD state to germination is accompanied by a decrease in sensitivity to ABA concomitant with an increase in sensitivity to GA [36].

### 2.3. Seed Water Uptake

**Figure 8:**
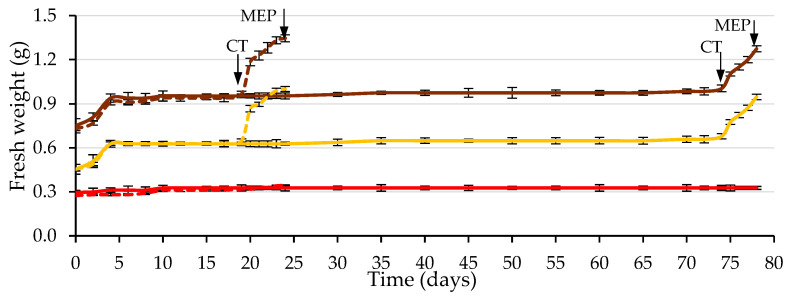
Time course of fresh weight (water uptake) during the germination test of caper seeds, considering seeds with testa (brown), the testa (red) and the endosperm containing the embryo (yellow). Seeds wetted with water are shown as solid lines, and those wetted with the 500 mg L^−1^ GA_3_ solution are shown as dashed lines (up to Day 19, solid and dashed lines practically overlap). The arrows indicate the dates on which the cracking of the testa (CT) and the piercing of the micropylar endosperm (MEP) occurred. Average values of four replicates of 10 seeds each. Vertical bars represent the standard error.

Figure 8 shows the typical triphasic increase in seed fresh weight during the germination process: the imbibition phase (Phase I), characterized by rapid water uptake; the lag phase (Phase II) with active metabolic activity and little water uptake; and radicle protrusion (Phase III), in which additional water uptake leads to fresh weight gain, cell enlargement and radicle protrusion [35].

This seed water uptake pattern coincides with previous studies on caper seeds [12]. It was observed that the testa fresh weight remained constant. Its water content remained constant, and therefore, the water increase only occurred in the endosperm and embryo, both using water and GA_3_. Phase II lasted from Day 5 to Day 19 when the GA_3_ solution was used, and from Day 5 to Day 74 when water was used. After testa cracking, the water absorption and swelling of the embryo and endosperm increased, and the radicle emerged through a hole in the ME. This was hypothetically due to tissue dissolution and/or increased growth potential of the emerging radicle.

### 2.4. Puncture Force Needed to Crack the Testa

Most biological materials (including diverse seed coats) are anisotropic, i.e., their mechanical properties differ for different load directions [17]. Although the forces to pierce and crack the testa would probably be different, they would probably be correlated; therefore, the puncture force was measured. Until the testa cracked (Day 20 and 75 for seeds wetted with GA_3_ and water, respectively), the puncture force required to pierce it remained practically constant, with average values (of those determined as indicated in Section 3) of 2.85 N in seeds wetted with GA_3_ and 2.86 N when wetted with water. Testa cracking occurred when the embryo’s growth potential overcame the resistance presented by the testa, producing cracking in the hilum-micropylar area and leaving the endosperm visible. The effect of the wetting solution on the puncture force required to pierce the testa at 0, 4 (corresponding to the maximum water uptake) and 20 (cracking of the testa in seeds wetted with GA_3_) days was statistically analyzed (Table 3).

There is a general trend in which an increase in the seed moisture content causes a decrease in fracture toughness [17], but in the present study, there was no weakening (*p* ≤ 0.05) of the testa with imbibition prior to cracking or differences (*p* ≤ 0.05) between the use of water and GA_3_. If the germination results (Table 1) and those of the puncture force required to pierce the testa (Table 3) were analyzed together, it was observed that there were no differences (*p* ≤ 0.05) between the use of water and the GA_3_ solution in terms of puncture force (2.88 and 2.85 N, respectively), but there was higher and faster germination (*p* ≤ 0.05) when GA_3_ was applied (*A* = 83%, *Gt*_50_ = 21 d) than when water was used (*A* = 22%, *Gt*_50_ = 77 d; Figure 4). The germination capacity of the seeds is the result of a balance between the physical restrictions imposed by the tissues surrounding the embryo (testa and endosperm) and the ability of the embryo to grow and protrude, allowing the elongation of the radicle [15]. Given these results and according to Davies et al. [35], gibberellins increase the growth potential of the embryo, increasing the swelling of the endosperm so that it leads to overcoming the mechanical resistance imposed by the testa and therefore to its cracking without weakening it. The increase in embryo growth potential required to crack the testa occurs after a long period after initial water imbibition and requires a signal from the embryo, which can be replaced by the addition of GA_3_ (that triggers the increase in both the content of endogenous GA and the GA/ABA ratio), which, in turn, shortens the period required to crack the testa.

### 2.5. Puncture Force Needed to Pierce the Endosperm

**Figure 9:**
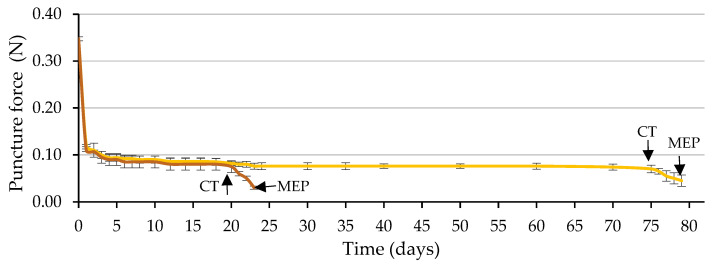
Time courses of endosperm weakening measured by the puncture force required to pierce the micropylar endosperm (ME) during the germination test, using a GA_3_ solution (500 mg L^−1^, in brown) or water (in yellow) to wet the substrate prior to ME piercing (MEP) by the radicle (3 days after testa cracking). The arrows indicate the dates on which the cracking of the testa (CT) and MEP occurred. Up to each CT, the values correspond to seeds with uncracked testa and from CT to seeds with cracked testa (prior to MEP by the radicle). Average values of four replicates of 10 seeds each. Vertical bars represent the standard error.

Figure 9 shows the time course of the puncture force required to pierce the ME. The required puncture force decreased drastically during the first day of imbibition (from 0.35 to 0.09 N, on average), using water and the GA_3_ solution, remaining practically constant and with identical values in both cases (approximately 0.08 N) until the testa cracked.

**Figure 10:**
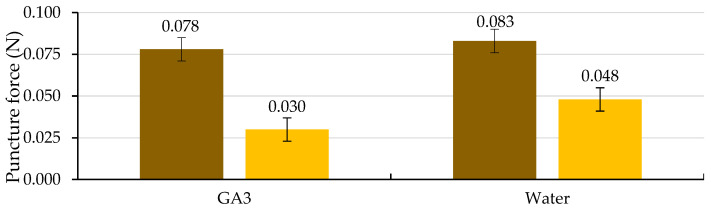
Analysis of the interaction of the analysis of variance in Table 4 between the cracking of the testa (uncracked testa (brown) and cracked testa (yellow)) and the solution used to wet the substrate on the puncture force required to pierce the micropylar endosperm at three days after the cracking of the testa. Average values of four replicates of 10 seeds each. Error bars represent the LSD (*p* ≤ 0.05).

Of the two factors analyzed, testa cracking and the solution used to wet the substrate, only the first factor significantly affected the puncture force (*p* ≤ 0.05; Table 4 and Figure 10). Following testa rupture, the puncture force to pierce the ME decreased to 0.030 N with the use of GA_3_ and 0.048 N with the use of water, not differing significantly (*p* ≤ 0.05; Table 4 and Figure 10). The radicle emerged through a hole in the ME, which was fundamentally a consequence of the weakening of the tissue and, to a lesser extent, of the increase in the growth potential of the emerging radicle, as stated in the literature [8], for tobacco seeds.

As in *Lepidium sativum* [20] seeds, during Phase II of germination, the embryo could exercise a signal to induce endosperm weakening; therefore, after testa cracking, GA application is not necessary to induce ME weakening. According to the general trend [17], ME weakening involves cell-wall loosening, cell separation, and programmed cell death to provide a decrease in localized ME tissue resistance, autolysis and finally, the formation of the ME hole required for radicle emergence. Unravelling how various aspects of seed germination are controlled, such as water uptake, dormancy and its release, seed viability and seedling vigor, as noted in a general way [17], will remain a major topic for research. Future studies will focus on unravelling the molecular mechanisms underlying caper seed germination.

## 3. Materials and Methods

### 3.1. Plant Material

The caper seed lot used in this study was obtained from adult plants grown in Llíria (39°38′54.2″ N, 0°37′3.5″ W; Valencia, Spain). The fruit collection was carried out in September 2020, and the experiments started on 1 April 2021. Once collected, the fruits were transferred to the laboratories of the Plant Production Department of the Universitat Politècnica de València (Valencia, Spain), where all the experiments were carried out. The seeds were extracted from ripe fruits collected on the day of their dehiscence and from fruits located in the anterior and posterior positions, as reported in previous studies [18]. These seeds were then disinfected with sodium hypochlorite (2 min) and rinsed in tap water. The mature seeds were selected through flotation in tap water, which is a standard method for separating viable from non-viable seeds [35]. Selected seeds were dried in the shade at room temperature (23–25 °C) for two weeks and then kept in closed airtight containers at 7 ± 0.5 °C in a domestic refrigerator (Beko, Beko Electronics España, Barcelona, Spain) until the tests started.

### 3.2. Viability and Germination Tests

According to the ISTA [27], the seed lot viability was determined by the tetrazolium test, as reported by the research team [3], using four replications of fifty seeds.

The germination test was carried out using intact seeds and seeds devoid of the testa (consisting of endosperm and embryo), which were obtained using tweezers and a scalpel. The germination tests were carried out following the between paper method (BP; [27]) in 9 cm Petri dishes. The substrate was wetted with ultra-pure water (Wasserlab G. R Type II analytical grade water system, hereinafter referred to as water) or a 500 mg L^−1^ GA_3_ solution (Semefil L, Nufarm Spain., Barcelona, Spain). To prevent fungal problems, in all cases, 2 g L^−1^ captan (Captan 50, Bayer) was added to the solution. Petri dishes were placed in a growth chamber (Zimbueze model, Seville, Spain) at 30 ± 1/20 ± 1 °C and 85 ± 1% relative humidity for a photoperiod of 12 h (cold white fluorescent tubes, Philips TL-D 36W/54, providing 81.1 μmol m^−2^ s^−1^) for a maximum of 120 days. The seeds were considered germinated when the radicle protruded through the structures surrounding it [9,35], and germinated seeds were eliminated from the Petri dish; evaluation was carried out every three days. Four replicates of 100 seeds each were used. For each replicate, the germination data were fitted to the logistic function [38,39], defined as a particular case of Richards’ function [40]: *G* = *A*/1 + e^(β−kt)^, where *G* is the cumulative germination (%), *A* represents the final germination percentage, t is the germination time (d; days) and *β* and *k* are function parameters used to determine the time (in d) required to reach 50% of *G* (*Gt*_50_ = *β/k*) and the mean relative cumulative germination rate (*k*/2, d^−1^). Considering the results obtained in the germination test, it was repeated including lower GA_3_ concentrations (0, 5, 50 and 500 mg L^−1^).

### 3.3. Hormone Content

Active GA (GA_1_, GA_3_, GA_4_ and GA_7_) and ABA contents were determined (in duplicate) in mature dry and imbibed seeds at 10, 20 and 75 (only for seeds wetted with water) days of the germination test, both in seeds with cracked and uncracked testa. For quantification, seeds were frozen in liquid nitrogen and stored at −80 °C until use at the Plant Hormone Quantification Service (IBMCP, Valencia, Spain). Then, they were ground into powder and suspended in an extraction solvent containing internal standards and mixed by shaking. The extracts were centrifuged, and the supernatant was dried in a vacuum evaporator. The dry residues were dissolved and passed through an Oasis HLB column [41]. The dried eluates were dissolved, and the active GA and ABA were separated using an auto sampler and reversed-phase UHPLC chromatography (2.6 µm Accucore RP-MS column, 100 mm length × 2.1 mm i.d.; Thermo Fisher Scientific, Waltham, MA, USA). GA and ABA were analyzed with a Q-Exactive mass spectrometer (Orbitrap detector; Thermo Fisher Scientific, Waltham, MA, USA) by targeted selected ion monitoring (SIM). The concentrations in the extracts were determined using embedded calibration curves and Xcalibur 4.0 and TraceFinder 4.1 SP1 programs.

### 3.4. Seed Water Uptake

To describe water uptake during the germination process, the fresh seed weight was periodically determined [35]. This periodicity varied throughout the experiment; measurements were carried out daily except between Day 20 and 75 in the seeds wetted with water (time with practically constant weight) when the measurements were taken every five days. Four replicates of 10 seeds each were weighed on an analytical balance (Sartorius, Model B 120S, Barcelona, Spain), following the methodology previously reported [12], including both the intact seeds and testa and the endosperm (including the embryo), separately.

### 3.5. Mechanical Resistance Determination

The puncture force necessary to pierce both the testa (in the hilum-micropylar area) and the empty endosperm (without the embryo) was measured after the indicated incubation period, following a similar methodology to that reported by Müller et al. [20] and Zhang et al. [42], using a digital fruit firmness tester (53205, TR Turoni, Foirli, Italy) with a 0.6 mm diameter metallic blunt-tipped needle. Empty endosperms were obtained by cutting the testa in half with a sharp scalpel and carefully extracting the endosperm, from which the embryo was carefully excised as in the excised-embryo test [35], leaving the ME intact, where the puncture force was measured. Four replicates of 10 seeds and 10 endosperms were measured for each treatment. The puncture force necessary to pierce the testa was measured daily during the first 20 days of the germination test, both in seeds wetted with GA_3_ solution or water and then every five days until 75 days only in seeds wetted with water. These dates, 20 and 75 d, correspond to the *Gt*_50_ obtained with the use of the GA_3_ solution or water to wet the substrate, respectively, since this time is when more seeds crack their testa per day, and mainly because after the cracking of the testa, it is not appropriate to determine its resistance. The puncture force necessary to pierce the ME was determined daily during the first 25 days and then every five days until 75 days in seeds wetted with water and was measured again daily afterwards. After 20 d for seeds wetted with GA_3_ and 75 d for those wetted with water, the puncture force necessary to pierce the ME corresponded to the seeds in which testa had naturally cracked and before the radicle protruded.

### 3.6. Data Analysis

The differences between the maximum and minimum values of the four replicates obtained in all tests met the tolerance levels [27]. The results were analyzed by multiway analyses of variance (ANOVA [43]). The percentage data were arcsin transformed before analysis. The normality distribution was analyzed by verifying the residual normal distribution [44] by the Shapiro–Wilk test [43]. A probability (*p*) ≤ 0.05 was considered significant. Mean separations were performed when appropriate using Fisher’s least significance difference (LSD test) at *p* ≤ 0.05.

## 4. Conclusions

Caper seeds have a nondeep physiological dormancy, which can be released by adding GA_3_ to the germination substrate. This addition triggers an increase in the content of endogenous GA and, to a greater extent, the GA/ABA ratio, advancing and increasing the germination percentage. Its germination consists of two temporally separated events: testa cracking and endosperm piercing. The cracking of the testa begins in the hilum-micropylar area, requires water absorption, and involves a signal from the embryo, which the addition of gibberellins can replace, increasing and advancing the percentage of seeds in which cracking occurs. This cracking is not due to a decrease in the mechanical constraint of the testa but rather to an increase in the growth potential of the embryo to overcome it. With 500 mg L^−1^ GA_3_, germination percentages similar to those obtained by eliminating the testa were obtained. After testa cracking, the radicle emerges through a hole in the micropylar endosperm. The puncture force necessary to pierce the micropylar endosperm decreased drastically during the first day of imbibition, remaining practically constant until testa cracking, decreasing afterwards, regardless of the addition or not of gibberellins.

## Figures and Tables

**Table 1:** Effect of the presence of the testa and the solution used to wet the substrate on the final germination (*A*, %), the number of days needed to reach 50% of the final germination (*Gt_5_*_0_, d) and the relative average rate of accumulated germination (*k*/2, d^−1^). Average values of four replicates.

	*A*	*Gt*_50_	*k*/2
Testa (T)			
With testa	52.78 b	49.06 a	0.113
Without testa	81.61 a	13.42 b	0.126
Solution (S)			
Water	54.16 b	43.24 a	0.109
GA_3_	80.22 a	19.25 b	0.130
Analysis of variance			
Factors (degrees of freedom)	% Sum of squares		
T (1)	30.05 **	40.01 **	0.64 NS
S (1)	24.55 **	18.12 **	1.52 NS
T × S (1)	44.43 **	32.43 **	29.52 NS
Residual (12)	0.97	9.44	68.33
Standard deviation (^+^)	3.00	9.99	0.08

Different letters in the same column within each factor indicate significant differences (*p* ≤ 0.05) according to the LSD test. **: significance level *p* ≤ 0.01, NS: not significant. (^+^) The standard deviation was calculated as the square root of the residual mean square.

**Table 2:** Effect of the presence of the testa and the concentration of the GA_3_ solution used to wet the substrate (0, 5, 50 and 500 mg L^−1^) on the final germination (*A*, %), the number of days needed to reach 50% of the final germination (*Gt*_50_, d) and the relative average rate of accumulated germination (*k*/2, d^−1^). Average values of four replicates.

	*A*	*Gt*_50_	*k*/2
Testa (T)			
With testa	32.69 b	63.86 a	0.247 b
Without testa	82.07 a	3.37 b	1.032 a
GA_3_ Concentration (C)			
0	50.89 b	39.21 a	0.205 b
5	52.30 b	40.06 a	0.990 ab
50	47.96 b	40.36 a	1.219 a
500	78.38 a	15.00 b	0.145 b
Analysis of variance			
Factors (Degrees of freedom)	% Sum of squares		
T (1)	52.00 **	77.30 **	15.14 *
C (3)	12.74 **	9.78 **	21.91 *
T × C (3)	33.57 **	11.92 **	13.12 NS
Residuals (24)	1.68	1.00	49.83
Standard deviation (^+^)	5.12	15.3	0.8

Different letters in the same column within each factor indicate significant differences (*p* ≤ 0.05) according to the LSD test. ** (*): significance level *p* ≤ 0.01 (0.05), NS: not significant. (^+^) The standard deviation was calculated as the square root of the residual mean square.

**Table 3:** Effect of the imbibition period (days from the beginning of the germination process) and the solution used to wet the germination substrate in the germination test on the puncture force necessary to pierce the testa at 0, 4 and 20 days after the start of the germination test. Average values of four replicates of 10 seeds each.

	Puncture Force (N)
Imbibition period (P)	
0	2.83
4	2.87
20	2.89
Solution (S)	
Water	2.88
GA_3_	2.85
Analysis of variance	
Factors (Degrees of freedom)	% Sum of squares
P (2)	4.29 NS
S (1)	1.31 NS
P × S (2)	1.93 NS
Residuals (18)	92.47
Standard deviation (^+^)	0.13

NS: Not significant at *p* ≤ 0.05. (^+^) The standard deviation was calculated as the square root of the residual mean square.

**Table 4:** Effect of the testa cracking and the solution used to wet the germination substrate in the germination test on the puncture force required to pierce the micropylar endosperm at three days after testa cracking. Mean values of 4 replicates of 10 seeds each.

	Puncture Force (N)
Testa (T)	
Uncracked	0.080 a
Cracked	0.039 b
Solution (S)	
Water	0.065
GA_3_	0.054
Analysis of variance	
Factors (Degrees of freedom)	% Sum of squares
T (1)	73.23 **
S (1)	5.45 NS
T × S (1)	1.68 NS
Residual (12)	19.64
Standard deviation (^+^)	0.01

Different letters within each factor indicate significant differences (*p* ≤ 0.05) according to the LSD test. **: significance level *p* ≤ 0.01, NS: not significant. (^+^) The standard deviation was calculated as the square root of the residual mean square.

## Data Availability

Not applicable.
